# Nerve Demyelination Increases Metabotropic Glutamate Receptor Subtype 5 Expression in Peripheral Painful Mononeuropathy

**DOI:** 10.3390/ijms16034642

**Published:** 2015-03-02

**Authors:** Miau-Hwa Ko, Yu-Lin Hsieh, Sung-Tsang Hsieh, To-Jung Tseng

**Affiliations:** 1 Department of Anatomy, College of Medicine, China Medical University, Taichung 40402, Taiwan; E-Mail: mhko@mail.cmu.edu.tw; 2 Department of Anatomy, School of Medicine, College of Medicine, Kaohsiung Medical University, Kaohsiung 80708, Taiwan; E-Mail: ylhsieh@kmu.edu.tw; 3 Department of Anatomy and Cell Biology, National Taiwan University College of Medicine, Taipei 10051, Taiwan; E-Mail: shsieh@ntu.edu.tw; 4 Department of Neurology, National Taiwan University Hospital, Taipei 10002, Taiwan; 5 Department of Anatomy, Institute of Medicine, Chung Shan Medical University, Taichung 40201, Taiwan; 6 Department of Medical Education, Chung Shan Medical University Hospital, Taichung 40201, Taiwan

**Keywords:** chronic constriction injury, peripheral hypersensitivities, metabotropic glutamate receptor subtype 5 (mGluR5), 2-methyl-6-(phenylethynyl)-pyridine (MPEP), cutaneous denervation, nerve demyelination

## Abstract

Wallerian degeneration or nerve demyelination, arising from spinal nerve compression, is thought to bring on chronic neuropathic pain. The widely distributed metabotropic glutamate receptor subtype 5 (mGluR5) is involved in modulating nociceptive transmission. The purpose of this study was to investigate the potential effects of mGluR5 on peripheral hypersensitivities after chronic constriction injury (CCI). Sprague-Dawley rats were operated on with four loose ligatures around the sciatic nerve to induce thermal hyperalgesia and mechanical allodynia. Primary afferents in dermis after CCI exhibited progressive decreases, defined as partial cutaneous denervation; importantly, mGluR5 expressions in primary afferents were statistically increased. CCI-induced neuropathic pain behaviors through the intraplantar injections of 2-methyl-6-(phenylethynyl)-pyridine (MPEP), a selective mGluR5 antagonist, were dose-dependently attenuated. Furthermore, the most increased mGluR5 expressions in primary afferents surrounded by reactive Schwann cells were observed at the distal CCI stumps of sciatic nerves. In conclusion, these results suggest that nerve demyelination results in the increases of mGluR5 expression in injured primary afferents after CCI; and further suggest that mGluR5 represents a main therapeutic target in developing pharmacological strategies to prevent peripheral hypersensitivities.

## 1. Introduction

Spinal nerve compression like sciatica in humans is a chronic nervous disease, which is accompanied with neuropathic pain. These obvious painful symptoms often occur in distal extremities, and include hypersensitivities to noxious stimuli (hyperalgesia), innocuous stimuli (allodynia) and spontaneous pain [1,2]. Several animal models of peripheral nerve injury resulting from nerve compression have been established, such as chronic constriction injury (CCI), partial sciatic nerve lesion (PSNL) and spinal nerve ligation (SNL) [3,4,5]. Most of these surgical models are developed for assessing pain-related behaviors in rats, which are equivalent to those seen in humans [6].

Primary afferents from dorsal root ganglia (DRG) neurons are classified into distinct fiber groups corresponding to their cytoskeletal organization and functions. It is known that myelinated Aβ fibers convey the light touch from mechanical stimuli whereas myelinated Aδ fibers conduct as mechanonociceptors and unmyelinated C fibers are primarily nociceptors [7]. The assessment of myelinated Aδ and unmyelinated C fibers in skin is examined by calculating the density of intraepidermal nerve fibers (IENFs) [8,9,10]. Furthermore, the decreased density of IENFs indicating the partial cutaneous denervation after CCI is a major requirement for developing the peripheral hypersensitivities. Recently, several studies focus on these primary afferents in dermis, described as subepidermal nerve fibers (SENFs) in human painful neuropathies [11,12,13]. Thus, the quantitation of SENFs distribution provides a novel strategy to evaluate the alterations of primary afferents in dermis, especially containing myelinated Aβ fibers.

The biological effects of glutamate in nociceptive mechanisms are mainly mediated by two major glutamate receptors: Ligand-gated ionotropic glutamate receptors (iGluRs) and G protein-coupled metabotropic glutamate receptors (mGluRs) [14,15]. l-glutamate is a principal excitatory neurotransmitter and known to participate in nociceptive signaling pathways by interacting with mGluRs [16,17]. Earlier studies identify the group I mGluRs, including subtype 1 and 5 of mGluRs (mGluR1 and mGluR5), localized in primary afferents [18,19]. Additionally, the role of mGluR5 in inflammatory pain is supposed by coupling to inositol phosphate metabolism to modulate the neuronal excitability and synaptic transmission [15,20,21]. Pharmacological evidence in periphery further suggests that the mGluR5 antagonist, but not the mGluR1 antagonist, is responsible for attenuating hyperalgesia following the inflammation pain and craniofacial muscle pain [19,22]. Nevertheless, no report has completely investigated mGluR5 expression in primary afferents or assessed the peripheral effects of mGluR5 antagonist after CCI.

Multiple mechanisms resulting from sciatic nerve injury demonstrate the nerve demyelination, ectopic discharge and macrophage infiltration are closely related to the development of neuropathic pain behaviors [23,24]. Myelinated A fibers at the distal CCI stumps of sciatic nerve undergoing nerve demyelination increase ectopic discharges, which are considered as injury-induced electrophysiological characteristics [25,26]. Moreover, the redistributions of voltage-gated sodium channels at node of Ranvier after surgical nerve decompression have confirmed a role in relieving peripheral hypersensitivities [27]. Therefore, we performed CCI in rats to (1) evaluate the temporal changes of thermal hyperalgesia and mechanical allodynia; (2) estimate the possible changes of SENFs in dermis after nerve compression; (3) assess the functional effects of mGluR5 by intraplantar injections with 2-methyl-6-(phenylethynyl)-pyridine (MPEP), a selective mGluR5 antagonist; and (4) clarify mGluR5 expressions along the distal CCI stumps of sciatic nerve and confirm mGluR5 localization by the means of double-labeled immunofluorescence.

## 2. Results

### 2.1. Neuropathic Pain Behaviors Following CCI

**Figure 1:**
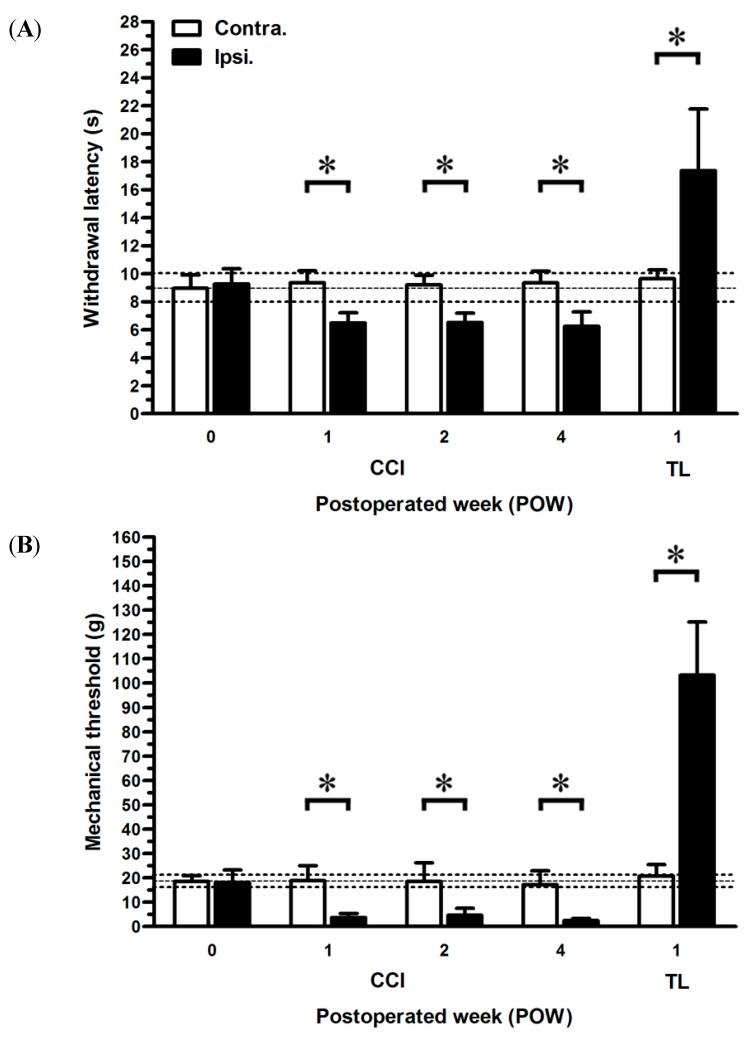
Effects of nerve compression injury on the temporal changes of neuropathic pain behaviors. The sequential changes of painful behaviors were shown in (**A**) thermal hyperalgesia and (**B**) mechanical allodynia. The thermal threshold of noxious radial heat was defined as withdrawal latency (s) and the degree of mechanical allodynia was expressed as the mechanical threshold (g) to the innocuous Von Frey filaments. Behavioral assessments were expressed as the mean ± standard deviation (SD) (*n* = 5 at each time points after chronic constriction injury (CCI), *n* = 5 at post-operated week (POW) 1 after tight ligation (TL)). Each bar of values depicted in the contralateral sides (Contra., open bars) and ipsilateral sides (Ipsi., filled bars). Student’s *t* test was applied to examine the differences between the contralateral and ipsilateral sides at the same time points. Two-way repeated measures ANOVA was also performed following Bonferroni’s *post-hoc* test. * *p* < 0.05, indicated as a significant difference.

The decreasing thresholds of noxious heat stimuli (thermal hyperalgesia) and innocuous stimuli (mechanical allodynia) exhibited a similar pattern after CCI (Figure 1). Withdrawal latencies at post-operated week (POW) 0 presented comparable results between both sides of rats (8.96 ± 0.95 s in the ipsilateral sides, 9.28 ± 1.08 s in the contralateral sides, *p* > 0.05) (Figure 1A). Hence, these values in the contralateral sides were as control to compare those in the ipsilateral sides at each time point. CCI in rats developed the painful responses within one week, such as everting and clenching the injured hindlimb, and even sudden licking the hindpaw. During the entire experimental period, withdrawal latencies showed persistant reductions in the ipsilateral sides of CCI (6.49 ± 0.72 s at POW 1, 6.51 ± 0.67 s at POW 2, and 6.25 ± 1.02 s at POW 4, *p* < 0.05, respectively). Conversely, tight ligation (TL) in rats revealed the increasing thresholds of withdrawal latency representing the thermal hypoalgesia at POW 1. (17.37 ± 4.39 s in the ipsilateral sides, *p* < 0.05). Before nerve compression injury, mechanical thresholds were parallel between both sides of rats (18.06 ± 5.15 g in the ipsilateral sides, 18.58 ± 2.33 g in the contralateral sides, *p* > 0.05) (Figure 1B). Through POW 1 to 4, the ipsilateral sides of CCI significantly reduced the values of mechanical threshold (3.66 ± 1.70 g at POW 1, 4.66 ± 2.87 g at POW 2, and 2.54 ± 0.80 g at POW 4, *p* < 0.05, respectively). The decreased responses to light touch were measured at POW 1 in the ipsilateral sides of TL (103.25 ± 24.85 g, *p* < 0.05).

### 2.2. The Degeneration of Neurofilament 200 (NF-200)-Immunoreactive (IR) SENFs in Dermis after CCI

**Figure 2:**
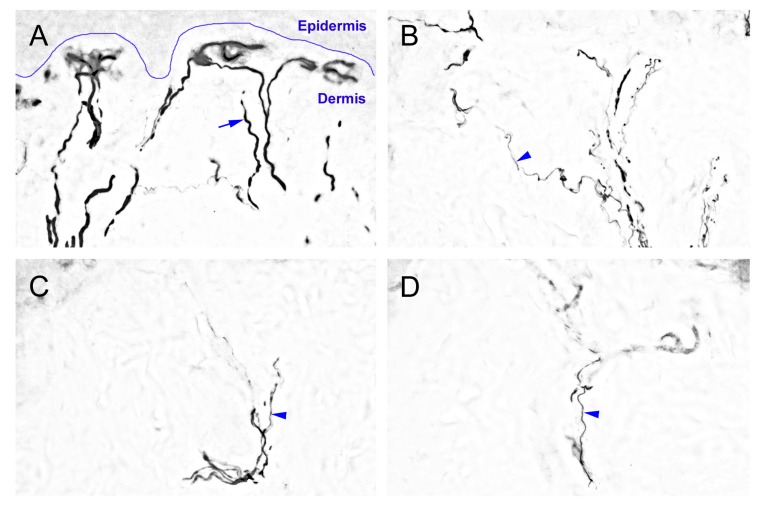

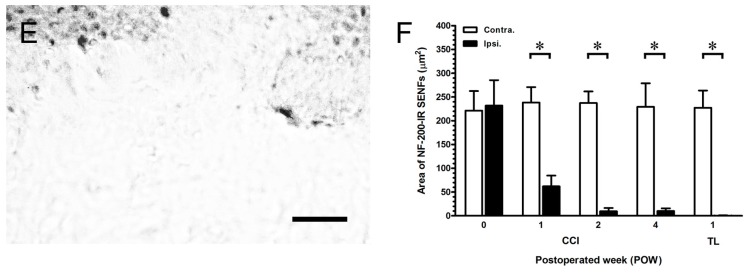
Neurofilament 200 (NF-200)-immunoreactive (IR) subepidermal nerve fibers (SENFs) in dermis under the influences of nerve compression injury. The ipsilateral sides of footpad skin from (**A**) sham-operated surgery, (**B**–**D**) CCI, and (**E**) TL were immunostained with antibody against NF-200. (**A**) Normal NF-200-IR SENFs exhibited abundant thick dermal fiber trunks (blue arrow), horizontally terminated around the epidermal-dermal junction (blue curve); Sequential changes of dermal expression after CCI were shown at (**B**) POW 1, (**C**) POW 2 and (**D**) POW 4, and thin NF-200-IR SENFs were revealed with irregular linear forms (blue arrowhead); (**E**) TL induced the complete fiber loss of NF-200-IR SENFs in dermis at POW 1. Scale bar = 25 μm; and (**F**) The areas of NF-200-IR SENFs were quantified as the mean ± SD (*n* = 5 at each time points after CCI, *n* = 5 at POW 1 after TL). Each bar of values showed in the contralateral sides (Contra., open bars) and ipsilateral sides (Ipsi., filled bars). Student’s *t* test was applied to examine the differences between the Contra. and Ipsi. at the same time points. * *p* < 0.05, indicated as a significant difference.

We evaluated morphological evidence with antibodies against NF-200 to understand the effects of nerve compression injury on myelinated A fibers in dermis (Figure 2). Abundant NF-200-IR SENFs formed the thick dermal fiber trunks, horizontally terminated around the epidermal-dermal junction with typical rod-like blunt ends in sham-operated surgery (Figure 2A). At POW 1 after CCI, thick NF-200-IR SENFs dramatically decreased having the occurrence of fragmented profiles (Figure 2B). All the thick NF-200-IR SENFs almost disappeared and only a little of thin NF-200-IR SENFs presented from POW 2 to 4 (Figure 2C,D). Compared with the surgery of TL, there was a complete loss of NF-200-IR SENFs at POW 1 (Figure 2E). The changes of immunohistochemical pattern in dermis were verified by the quantitation of area of NF-200-IR SENFs with µm^2^ (Figure 2F). After sham-operated surgery, values were equivalent between both sides (231.74 ± 53.56 µm^2^ in the ipsilateral sides, 221.46 ± 41.00 µm^2^ in the contralateral sides, *p* > 0.05). In the ipsilateral sides of CCI, values were significantly reduced at POW 1 and intense depletions lasted from POW 2 to 4 (61.84 ± 22.69 µm^2^ at POW 1, 9.54 ± 6.90 µm^2^ at POW 2, and 10.11 ± 5.69 µm^2^ at POW 4, *vs.* contralateral sides, *p* < 0.05, respectively). After TL at POW 1, values showed the diminished deceases in the ipsilateral sides (0.32 ± 0.83 µm^2^, *vs.* contralateral sides, *p* < 0.05).

### 2.3. CCI-Induced the Significant Loss of Dermal Calcitonin Gene-Related Peptide (CGRP)-IR SENFs

**Figure 3:**
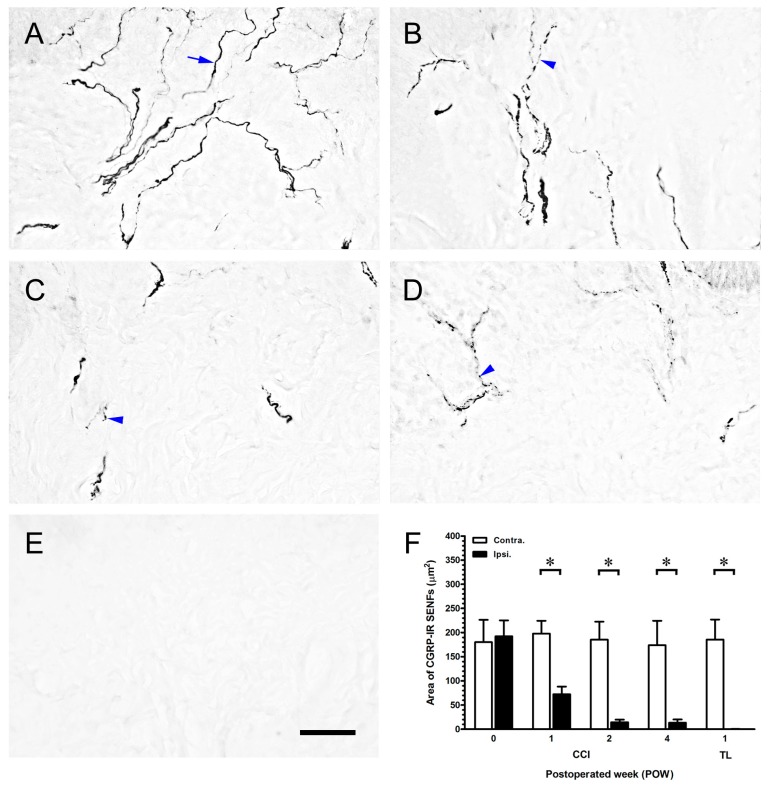
Influences of nerve compression injury on calcitonin gene-related peptide (CGRP)-IR SENFs in dermis. The ipsilateral sides of footpad skin from (**A**) sham-operated surgery, (**B**–**D**) CCI, and (**E**) TL were immunostained with antibody against CGRP. (**A**) Irregular linear CGRP-IR SENFs in normal dermis were towards the epidermal-dermal junction (blue arrow); Temporal changes of dermal expression were exposed at (**B**) POW 1, (**C**) POW 2 and (**D**) POW 4, and CGRP-IR SENFs had obvious beaded and swollen fragments after CCI (blue arrowhead); (**E**) Entire CGRP-IR SENFs loss was presented after TL at POW 1. Scale bar = 25 μm; and (**F**) The areas of CGRP-IR SENFs were quantified as the mean ± SD (*n* = 5 at each time points after CCI, *n* = 5 at POW 1 after TL). Each bar of values showed in the contralateral sides (Contra., open bars) and ipsilateral sides (Ipsi., filled bars). Student’s *t* test was applied to examine the differences between the Contra. and Ipsi. at the same time points. * *p* < 0.05, indicated as a significant difference.

To illustrate the alterations of peptidergic SENFs in dermis by nerve compression injury, sections were immunostained with antibodies against CGRP (Figure 3). In sham-operated surgery, irregular linear CGRP-IR SENFs were towards the epidermal-dermal junction to form the subepidermal nerve plexus (Figure 3A). After CCI, these individual CGRP-IR SENFs had obvious beaded appearances and swollen fragments from POW 1 to 4 (Figure 3B–D). There also was a complete loss of CGRP-IR SENFs following TL at POW 1 (Figure 3E). The quantitations demonstrated the temporal changes of area of CGRP-IR SENFs on the sections of footpad skin (Figure 3F). The values of sham-operated surgery in the ipsilateral sides (192.11 ± 33.08 µm^2^) corresponded to those in the contralateral sides (180.00 ± 46.20 µm^2^, *p* > 0.05). Values considerably decreased in the ipsilateral sides of CCI at POW 1 and relative reductions began from POW 2 to 4 (72.23 ± 15.87 µm^2^ at POW 1, 14.54 ± 5.35 µm^2^ at POW 2, and 13.37 ± 6.90 µm^2^ at POW 4, *vs.* contralateral sides, *p* < 0.05, respectively). Following TL at POW 1, values in the ipsilateral sides confirmed the observations in the entire loss of CGRP-IR SENFs (0.19 ± 0.42 µm^2^, *vs.* contralateral sides, *p* < 0.05).

### 2.4. Intensely Increased mGluR5-IR SENFs in Dermis Following CCI

**Figure 4:**
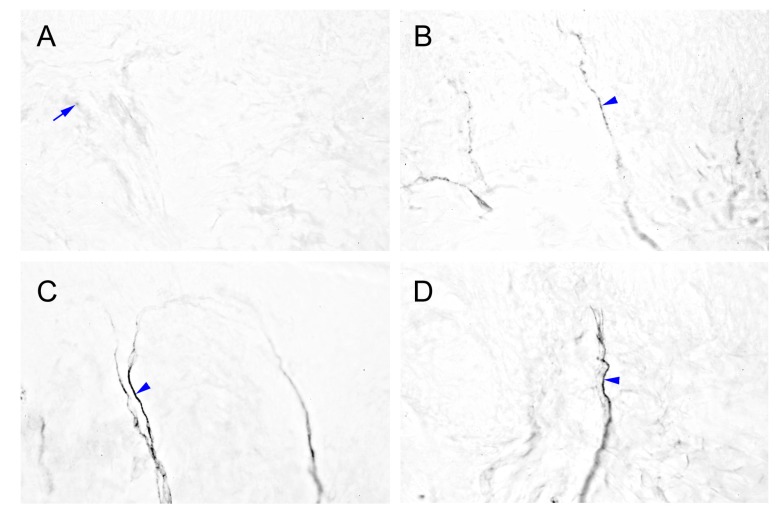

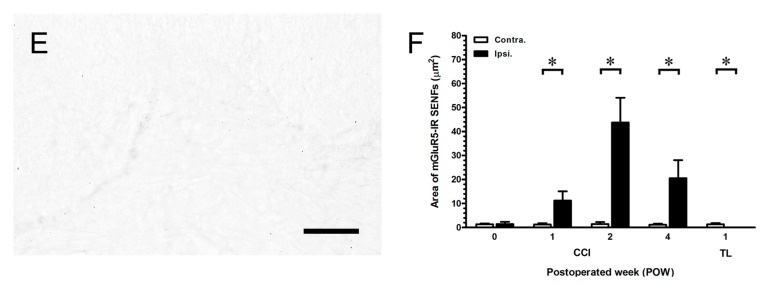
Cutaneous distributions of metabotropic glutamate receptor subtype 5 (mGluR5)-IR SENFs following the nerve compression injury. The ipsilateral sides of footpad from (**A**) sham-operated surgery, (**B**–**D**) CCI, and (**E**) TL were immunostained with antibody against mGluR5. (**A**) mGluR5-IR SENFs were slightly expressed in normal dermis (blue arrow); (**B**) Beginning from POW 1 after CCI, mGluR5-IR SENFs revealed the granular appearances toward the epidermal-dermal junction (blue arrowhead); (**C**,**D**) The continued dense forms of mGluR5-IR SENFs were presented from POW 2 to 4 (blue arrowhead); (**E**) Sciatic nerve with TL at POW 1 was lacking the distribution of mGluR5-IR SENFs. Scale bar = 25 μm; and (**F**) Quantitative analyses on the areas of mGluR5-IR SENFs were expressed as the mean ± SD (*n* = 5 at each time points after CCI, *n* = 5 at POW 1 after TL). Each bar of values showed in the contralateral sides (Contra., open bars) and ipsilateral sides (Ipsi., filled bars). Student’s *t* test was applied to examine the differences between the Contra. and Ipsi. at the same time points. * *p* < 0.05, indicated as a significant difference.

We examined the immunohistochemical staining to illustrate a possible change of mGluR5-IR SENFs in dermis after nerve compression injury (Figure 4). These SENFs showed slight mGluR5 expression in the normal dermis of sham-operated surgery (Figure 4A). Beginning from POW 1 after CCI, increased mGluR5-IR SENFs presented granular appearances toward the epidermal-dermal junction (Figure 4B). The dense forms of mGluR5-IR SENFs were still significantly observed from POW 2 to 4 (Figure 4C,D). Remarkably, the existence of mGluR5-IR SENFs was not detected after TL at POW 1 (Figure 4E). The morphological changes of mGluR5-IR SENFs in dermis were confirmed by quantitative comparisons (Figure 4F). The dermal areas of mGluR5-IR SENFs in both sides of sham-operated surgery were comparatively low (1.41 ± 0.95 µm^2^ in the ipsilateral sides, 1.39 ± 0.34 µm^2^ in the contralateral sides, *p* > 0.05). CCI in rats induced considerable increases of values from POW 1 to 2 (11.21 ± 3.86 µm^2^ at POW 1, 43.73 ± 10.33 µm^2^ at POW 2, *vs.* contralateral sides, *p* < 0.05, respectively). The values starting from POW 2 to 4 were moderately reduced in the ipsilateral sides of CCI (20.52 ± 7.52 µm^2^ at POW 4, *vs.* contralateral sides, *p* < 0.05). The diminished values after TL illustrated the loss of mGluR5-IR SENFs at POW 1 (0.02 ± 0.03 µm^2^, *vs.* contralateral sides, *p* < 0.05).

### 2.5. MPEP Attenuated the Neuropathic Pain Behaviors by Intraplantar Injections

**Figure 5:**
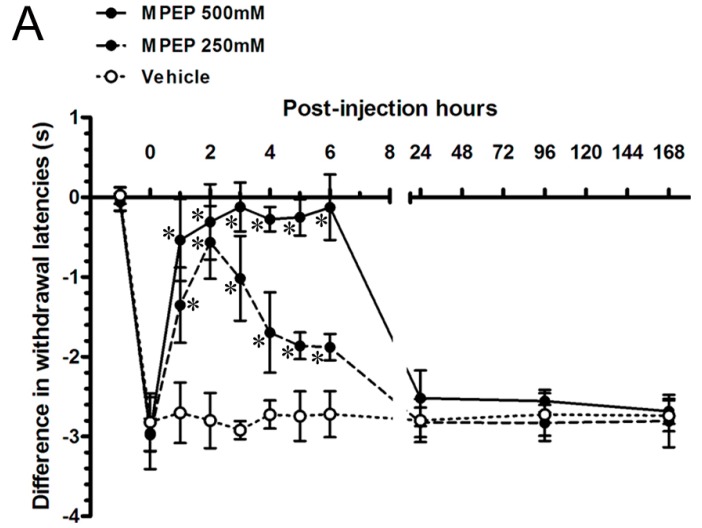

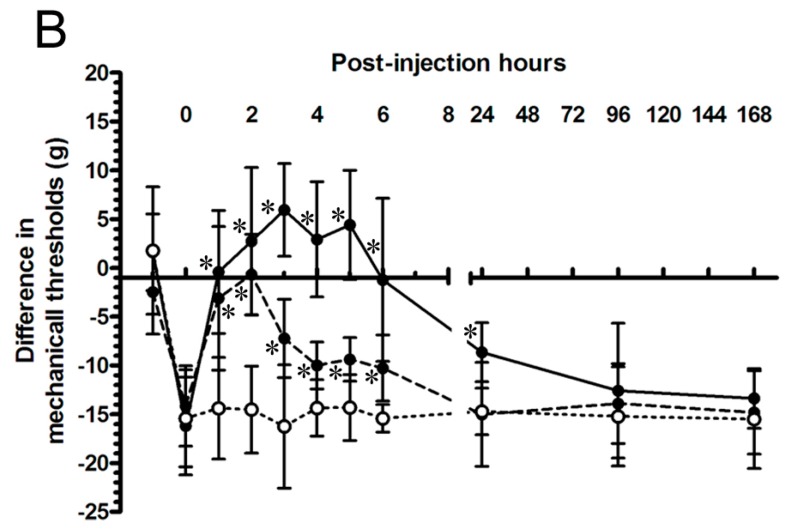
The pharmacological effects of MPEP on the CCI-induced peripheral hypersensitivities. The effects of MPEP, a selective mGluR5 antagonist, were applied on CCI by intraplantar injections to assess the (**A**) thermal hyperalgesia and (**B**) mechanical allodynia following post-injection hour (PIH). The differences in withdrawal latency and mechanical threshold were calculated by subtracting the responses of contralateral side from the responses of ipsilateral side and presented as the mean ± SD. The properties of MPEP were verified at the concentration of 500 mM (filled circles with solid line) and 250 mM (filled circles with dash line), and vehicle (open circles with dotted line) (*n* = 6 per group). Student’s *t* test was applied to examine the differences compared to vehicle injections at the same time points. One-way repeated measures ANOVA was also used to analyze the within-group differences following Dunnett’s multiple comparison tests. * *p* < 0.05, indicated as a significant difference.

To test the hypothesis whether increased mGluR5-IR SENFs in dermis contributed to develop neuropathic pain behaviors, we investigated the effects of MPEP by intraplantar injections in rats after CCI. The differences in the withdrawal latency and withdrawal threshold were used to calculate between both sides of CCI to evaluate peripheral hypersensitivities (Figure 5). The differences in painful behaviors at POW 2 after CCI were recorded and defined as values at post-injection hours (PIH) 0. First, the vehicle injections showed the persisted values of difference in withdrawal latency through the experimental period from PIH 1 (−2.70 ± 0.38 s) to PIH 168 (−2.74 ± 0.19 s) (*vs.* PIH 0, *p* > 0.05, respectively). Thus, these values after vehicle injections at each time point were used to compare those after MPEP injections. Beginning from 500 mM MPEP injections, values rapidly intensified up and returned to normal from PIH 1 (−0.54 ± 0.51 s) to PIH 6 (−0.13 ± 0.41 s) (*p* < 0.05, respectively) (Figure 5A). Thermal hyperalgesia redeveloped from PIH 24 (−2.52 ± 0.35 s) through the period to PIH 168 (−2.68 ± 0.16 s) (*p* > 0.05, respectively). After 250 mM MPEP injections, values partially reversed at PIH 1 (−1.35 ± 0.47 s) and normalized at PIH 2 (−0.57 ± 0.46 s) (*p* < 0.05, respectively). The thermal responses gradually returned toward the direction of thermal hyperalgesia from PIH 3 (−1.02 ± 0.53 s) to PIH 6 (−1.88 ± 0.16 s) (*p* < 0.05, respectively). The degree of thermal hyperalgesia was measured from PIH 24 (−2.82 ± 0.19 s) to PIH 168 (−2.81 ± 0.33 s) similar to that before MPEP injections (*p* > 0.05, respectively). The alterations of difference in mechanical threshold after intraplantar MPEP injections followed the similar temporal patterns such as the dose-response effects of thermal hyperalgesia (Figure 5B), *i.e.*, measured values illustrated the complete relief of mechanical allodynia at PIH 2 after intraplantar MPEP injections (2.72 ± 7.55 g in 500 mM MPEP group, 0.69 ± 4.14 g in 250 mM MPEP group) (*p* < 0.05, respectively).

### 2.6. MGluR5-IR Fibers Increased along the Distal CCI Stumps of Sciatic Nerve

**Figure 6:**
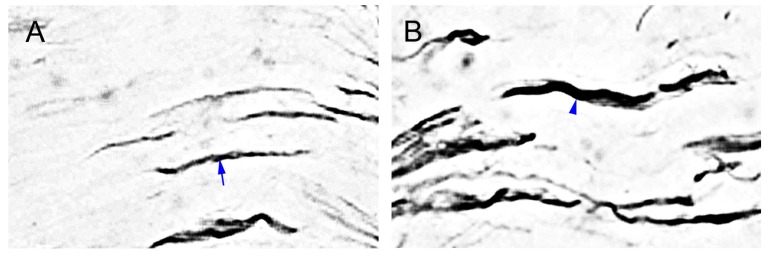

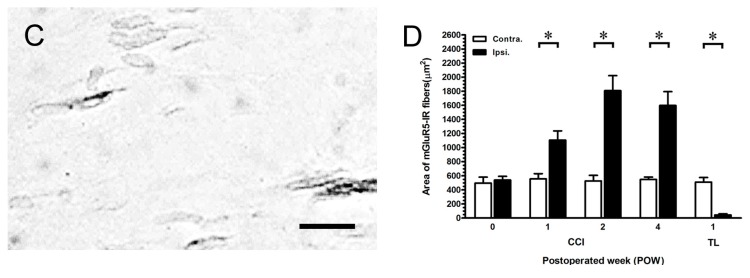
Distribution of mGluR5-IR fibers along the distal compression stumps of sciatic nerve. The sciatic nerves at POW 1 from the ipsilateral sides of (**A**) sham-operated surgery, (**B**) CCI, and (**C**) TL were immunostained with antibody against mGluR5. (**A**) The mGluR5-IR fibers typically expressed the dense linear appearances (blue arrow); (**B**) The more thick forms were presented in mGluR5-IR fibers (blue arrowhead); (**C**) The mGluR5-IR fibers were shown with the weakened occurrences. Scale bar = 25 μm; and (**D**) The areas of GluR5-IR fibers were quantified as the mean ± SD (*n* = 5 at each time points after CCI, *n* = 5 at POW 1 after TL). Each bar of values showed in the contralateral sides (Contra., open bars) and ipsilateral sides (Ipsi., filled bars). Student’s *t* test was applied to examine the differences between the Contra. and Ipsi. at the same time points. * *p* < 0.05, indicated as a significant difference.

We assessed the nerve stumps distal to nerve compression with antibodies against mGluR5 to confirm the altered distributions of mGluR5-IR SENFs in dermis (Figure 6). The sections of sciatic nerve showed the dense linear appearances in mGluR5-IR fibers after sham-operated surgery (Figure 6A). At POW 2, mGluR5-IR fibers at the distal CCI stumps revealed the more enhanced intensities (Figure 6B). In contrast, mGluR5-IR fibers after TL exhibited the weakened occurrences at POW 1 (Figure 6C). These mGluR5-IR fibers at the distal nerve stumps were quantified following a procedure to analyze the temporal changes after compression injury (Figure 6D). In sham-operated surgery, the areas of mGluR5-IR fibers between the both sides were almost the same (539.41 ± 51.26 µm^2^ in the ipsilateral sides, 496.16 ± 83.48 µm^2^ in the contralateral sides, *p* > 0.05). Resulting from CCI, values drastically increased from POW 1 to 2 (1105.35 ± 131.26 µm^2^ at POW 1, 1807.20 ± 214.58 µm^2^ at POW 2, *vs.* contralateral sides, *p* < 0.05, respectively). Then, values had the progressive decreases starting from POW 2 to 4 (1597.35 ± 196.31 µm^2^, *vs.* contralateral sides, *p* < 0.05). In the ipsilateral sides of TL, the reduced areas of mGluR5-IR fibers at POW 1 demonstrated the nearly complete fiber loss (45.13 ± 14.69 µm^2^, *vs.* contralateral sides, *p* < 0.05).

### 2.7. p75 Neurotrophin Receptor (p75NTR)-IR Reactive Schwann Cells Surrounded the mGluR5-IR Fibers at the Distal CCI Stumps of Sciatic Nerve

**Figure 7:**
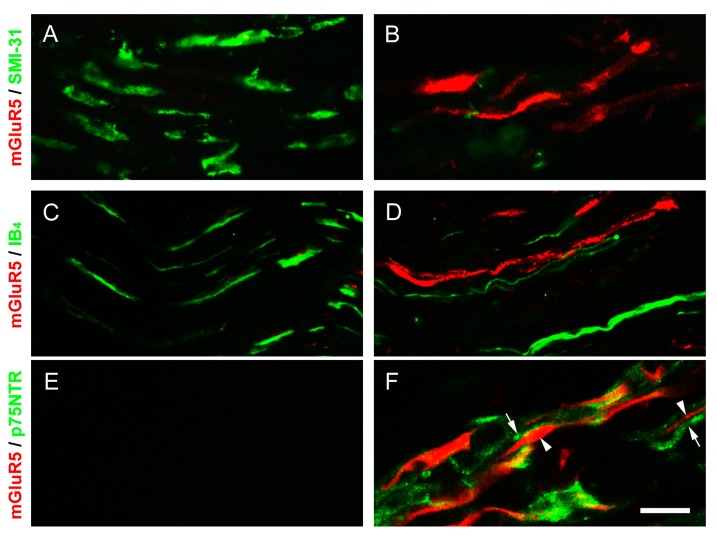
Double-labeled immunofluorescence along the distal CCI stumps of sciatic nerve. The mGluR5-IR fibers (red) at POW 2 in the ipsilateral sides of sciatic nerve were illustrated after (**A**,**C**,**E**) sham-operated surgery and (**B**,**D**,**F**) CCI. These merged images showed the intact fibers combined with antibody against (**A**,**B**) phosphorylated neurofilament (SMI-31) and (**C**,**D**) Griffonia (Brandeiraea) simplicifolia isolectin B4 (IB4) (green). Moreover, the localization of reactive Schwann cells was demonstrated with antibody against (**E**,**F**) p75 neurotrophin receptor (p75NTR) (green). p75NTR-IR reactive Schwann cells (arrows, in **F**) exhibited the irregular flattened appearance surrounding the mGluR5-IR fibers (arrowheads, in **F**). Scale bar = 25 μm.

In order to elucidate the localizations of mGluR5-IR fibers following CCI, we further used the double-labeled immunofluorescence to combine with the phosphorylated neurofilament (SMI-31), Griffonia (Brandeiraea) simplicifolia isolectin B_4_ (IB_4_), and p75NTR. These merged images showed their relative colocalizations at POW 2 (Figure 7). The phosphorylated neurofilaments in myelinated A fibers were expressed as SMI-31-IR fibers along the sciatic nerve in sham-operated surgery (Figure 7A). In contrast, the large amounts of SMI-31-IR fibers diminished and slightly colocalized with mGluR5-IR fibers after CCI (Figure 7B). IB_4_-IR fibers, known as the non-peptidergic fibers, presenting the discontinued linear pattern were observed in sham-operated surgery (Figure 7C). Partially decreased IB_4_-IR fibers after CCI did not show the significant colocalizations with mGluR5-IR fibers (Figure 7D). In addition, we examined the sections of sciatic nerve with antibodies against p75NTR to illustrate the changes of reactive Schwann cells resulting from nerve demyelination. In sham-operated surgery, thus, there were no obvious p75NTR-IR reactive Schwann cells along the sciatic nerve (Figure 7E). Conversely, we found these p75NTR-IR-reactive Schwann cells enlarged the irregular flattened appearances and surrounded increased mGluR5-IR fibers following CCI (Figure 7F). As present results, we suggest the CCI-induced increases of mGluR5 expression were along the demyelinated A fibers at the distal stumps of sciatic nerve.

## 3. Discussion

### 3.1. Different Degrees of Nerve Compression Used to Assess Behaviors

The animal models underlying partial nerve compression are important for developing neuropathic pain behaviors, including the CCI, PSNL, and SNL [3,4,5]. Combining our previous findings with these observations, CCI in rats reveals significant thermal hyperalgesia and mechanical allodynia and lasts at least for about three months [10]. Moreover, thermal hyperalgesia and mechanical allodynia are relieved by the redistributions of voltage-gated sodium channels after surgical nerve decompression [27]. Given the above results, TL in rats are compared to prove the obvious peripheral hyposensitivities and autotomy, such as those observed in complete sciatic nerve transection [28,29]. It is also known that a deficiency of nociceptive transmission is caused by Wallerian degeneration and a significant spontaneous pain is originated from traumatic neuroma [30,31,32]. In present study, we confirmed the partial nerve compression, but not complete nerve compression established an essential role in nociceptive transmission.

### 3.2. The Important Relationships between the Peripheral Hypersensitivities and Cutaneous Denervations

Human skin biopsy has developed as a valuable approach to estimate the pathological diagnosis of small fiber painful neuropathy [21,33,34]. Several clinical studies demonstrate the partial IENFs degeneration is a requirement for developing neuropathic pain behaviors [35,36,37]. The observations in CCI correspond to these results, when partial IENFs degeneration in footpad skin initiates peripheral hypersensitivities [8,9,10]. Recently, a few quantitative methods applied in human skin biopsy to estimate the degrees of myelinated A fiber degeneration in dermis after painful neuropathy [11,12,38]. As we have known, DRG neurons expressed NF-200 do not exhibit any noticeable neuronal loss and size shift after CCI. Moreover, NF-200 is limited in its ability to distinguish myelinated Aβ and Aδ fibers from DRG neurons. [39,40]. Thus, our current results suggested that CCI induced the significant decreases of NF-200-IR SENFs, indicating the both types of myelinated A fibers degeneration occurred in dermis.

In addition, the alterations of peptidergic substance P (SP)- and CGRP-IR IENFs have assessed their roles in painful behaviors following CCI and nerve decompression [8,9,10]. However, only a study in CCI observes the decreases of CGRP- and P2X3 receptor-IR SENFs in dermis and strongly suggests the CGRP-IR SENFs have better regenerative ability within one month [41]. Our present data was in conflict with these results and exhibited the partial loss of CGRP-IR SENFs even lasting for one month after CCI. TL in rats showed the comprehensive fiber loss in dermis within one week, including the NF-200- and CGRP-IR SENFs. In summary, the partial nerve denervation in dermis such as those observed in epidermis was an important foundation for developing neuropathic pain behaviors.

### 3.3. The Efficient Role of mGluR5 in Neuropathic Pain Behaviors

The G protein-coupled mGluRs are known to participate in nociceptive signaling pathways by coupling to inositol phosphate metabolism [16,17,18]. Group I mGluRs localized in the primary afferents of footpad skin are equal in their expression of all the small- and most medium-size DRG neurons [18,19]. In addition, the increases of mGluR5 expression in the lamina II of ipsilateral spinal cord, injured DRG neurons, and injured proximal stumps of sciatic nerve are supposed to modulate SNL-induced neuropathic pain behaviors [42]. Morphological evidence from this study indicated that large amounts of mGluR5-IR SENFs were presented in dermis following CCI. Comparatively, rats after TL showed the absence of mGluR5-IR SENFs in dermis based on complete fiber loss.

In association with the inhibitory effects of MPEP, our sequential evaluations in CCI further revealed the thermal hyperalgesia and mechanical allodynia were equally attenuated following intraplantar injections. The growing evidence illustrate the different methods of MPEP administration in CCI directly influence the painful behaviors. For instance, intracerebroventricular injection attenuates cold hyperalgesia [43]; intrathecal injection effectively decreases the development of thermal hyperalgesia, cold hyperalgesia, and transiently reduces mechanical hyperalgesia [44,45,46]; intraperitoneal injection increases the threshold of mechanical allodynia and cold hyperalgesia [43,47]. Interesting, intraplantar MPEP injection is ineffective in reversing CCI-induced cold hyperalgesia [43].

About the availability of MPEP in periphery, a study has proved its functional part in reducing the group I mGluR agonist-induced mechanical hyperalgesia [19]. Furthermore, the efficient role of mGluR5 is also confirmed by intraperitoneal MPEP injections in the animal models of SNL, inflammatory pain, and post-operative pain [22,32,42,48]. Another selective mGluR5 antagonist, SIB-1757, was given the full relief of thermal hyperalgesia by intrathecal and intraplantar injections in SNL; but the limited reversal of mechanical allodynia only by intrathecal injection [49].

### 3.4. The mGluR5 Localization in Periphery

Earlier reports demonstrate that there is not any noticeable pathological change at the proximal CCI stumps of sciatic nerve [7,50]. But at the distal CCI stumps, a few obscure results illustrate the more variable alterations of nerve degeneration. For example, myelinated A and unmyelinated C fibers display a parallel decrease along the distal CCI stumps of sciatic nerve [7,51,52]. Other studies illustrate the myelinated A fibers are observed the more serious damages at the distal nerve stumps after CCI [50,53,54]. Based on our observations with the existing results, we could not elucidate the degrees of nerve degeneration and need further detailed analysis on this issue.

Through the double-labeled immunofluorescent studies, however, we found the SMI-31- and IB_4_-IR fibers mostly decreased at the distal nerve stumps following CCI. Furthermore, these fibers did not show the significant colocalizations in combination with the antibody against mGluR5, indicating that mGluR5 was not expressed in intact primary afferents. A single cobra venom injection, and in periaxin-deficient mice, the dephosphorylation of neurofilaments, resulte in paranodal demyelination associated with painful behaviors [55,56]. Thus, our results in this study further demonstrated the increased p75NTR-IR-reactive Schwann cells exhibited in distal nerve stumps to an injury underwent an acute demyelination; whereas these injured myelinated A fibers increased mGluR5 expressions.

In addition, other possible mechanisms in neuropathic pain behaviors may be involved in: (1) the abolition of myelinated A fiber inhibition promotes the modulation of unmyelinated C fibers function [56]; and (2) the reactive Schwann cells after CCI release several inflammatory mediators, such as interleukin-1 β, interleukin-6, and tumor necrosis factor-alpha [57,58]. In the study of surgical nerve decompression, we have illustrated the redistribution of voltage-gated sodium channels along the distal CCI stumps is essential for pain-relief. [27]. Here, we demonstrated a critical step was to avoid demyelination to decrease mGluR5 expressions, which might provide a novel therapeutic approach for relieving the peripheral hypersensitivities associated with CCI.

## 4. Experimental Section

### 4.1. Animals

Adult male Sprague-Dawley rats, weighing 250–300 g, were used in these experiments. These rats were placed in a temperature- and humidity-controlled room with a 12 h light/dark cycle. Food and water were available *ad libitum.* All the procedures were conducted in accordance with the ethical guidelines set up by the International Association for the Study of Pain (IASP) on the use of laboratory animals in the experimental research and the protocol was approved by the Animal Committee of National Taiwan University College of Medicine, Taipei, Taiwan [59,60].

### 4.2. Surgeries

CCI was performed in rats following the established surgical procedures [3]. Briefly, under pentobarbital anesthesia (60 mg/kg, i.p.), the right sciatic nerve was exposed at mid-thigh level by freeing the adhering fascia between the gluteus and biceps femoris muscles. Four ligatures (4/0 chromic gut) were tied loosely around the sciatic nerve at 1-mm intervals above the nerve’s trifurcation. The ligatures constricted only about 1/3–1/4 of diameter of nerve and produced a brief twitch in the muscle around the exposure. To compare the different degrees of nerve compression, we also performed the TL by similar surgical procedures of CCI requiring a ligature compactly around the sciatic nerve [29]. The surgical side was defined as the ipsilateral side with its control side as the contralateral side in the following analyses.

### 4.3. Neuropathic Pain Behaviors

#### 4.3.1. Thermal Hyperalgesia

We evaluated the thermal hyperalgesia with a Hargreaves-type analgesiometer (Ugo Basile, Comerio-Varese, Italy) [3]. A radiant heat source (a halogen projector lamp, 50 W, 8 V) was placed directly beneath the plantar surface of hindpaw. Withdrawal latency was automatically measured as the time elapsed from the onset of radiant heat stimulation to the withdrawal of hindpaw. Each hindpaw was alternatively tested seven times with a minimal interval of 5 min between measurements. The values of last five consecutive measurements were used for the analysis and averaged as a thermal threshold.

#### 4.3.2. Mechanical Allodynia

Mechanosensitivity was determined by measuring the withdrawal thresholds to a series of calibrated Von Frey filaments (Senselab aesthesiometer, Somedic Sales AB, Stockholm, Sweden) according to an up-and-down method [27]. The examiner touched the plantar surface of hindpaw with a filament until the bending angle reached 45° with a brisk withdrawal or paw flinching was noted, which was considered a positive response. Mechanical threshold was defined as the minimal force (g) initiating a withdrawal response.

### 4.4. Immunohistochemistry of Footpad Skin

#### 4.4.1. Procedures

At the end of experiments, rats were anesthetized with isoflurane and sacrificed by the intracardiac perfusion of 4% ice-cold paraformaldehyde in 0.1M phosphate buffer (PB) at pH 7.4. Footpads were fixed for another 6 h and then changed to 0.1 M PB for storage. After a thorough rinsing in PB, samples were cryoprotected with 30% sucrose in 0.1 M PB overnight. The footpad perpendicular to epidermis was sectioned at 30 μm on a sliding microtome (HM440E; Microm, Walldorf, Germany), labeled sequentially, and stored at −20 °C. The sections for immunohistochemistry were treated with 0.5% Triton X-100 in 0.5 M Tris buffer (Tris), pH 7.6, for 30 min and processed for immunostaining. Briefly, the sections were quenched with 1% H_2_O_2_ in methanol and blocked with 5% normal goat serum in 0.5% nonfat dry milk/Tris. The sections were incubated with respective primary antiserum at 4 °C overnight. All of these antisera including: (1) rabbit polyclonal NF-200 (1:1000; Sigma Chemicals, St. Louis, MO, USA); (2) rabbit polyclonal CGRP (1:1000; Chemicon, Temecula, CA, USA); and (3) rabbit polyclonal metabotropic glutamate receptor subtype 5 (mGluR5) (1:500; Millipore, Billerica, MA, USA). After rinsing in Tris, the sections were incubated with the biotinylated goat anti-rabbit IgG (1:100; Jackson ImmunoResearch Laboratories, West Grove, PA, USA) for 1 h and the avidin-biotin complex horseradish peroxidase reagent (Vector Laboratories, Burlingame, CA, USA) for another hour. Reaction products were demonstrated with the 3,3'-diaminobenzidine (DAB, Sigma Chemicals).

#### 4.4.2. Quantitation

Standard procedure was following a protocol modified from a previously published method [11]. Briefly, we first photographed the high-definition monochrome images under an Olympus microscope (BH2; Olympus, Tokyo, Japan) with a digital camera at a magnification of 100×. Based on these images, the dermal area was defined as the area below the dermal-epidermal junction at a depth of 200 μm. All the areas-of-interest in dermis were measured with Adobe Photoshop Elements 2.0 (Adobe Systems, San Jose, CA, USA) and edited above the threshold levels to eliminate the background noise. Area-of-interest was measured in pixels and then transformed to μm^2^ according to the relationships between the pixel size and magnification. There were six footpad sections for each rat, and the investigator carrying out these measurements was blinded to the treatment of rats.

### 4.5. Pharmacological Intervention

#### 4.5.1. Drugs

Ten percent ethanol, 10% Tween and 80% saline were mixed as the vehicle solution. MPEP, a selective antagonist of mGluR5, was purchased from Tocris Cookson (Ballwin, MO, USA) and used in all experiments as its hydrochloride salt (*M*w = 229.7) [61]. MPEP were applied at the final concentration of 500 and 250 mM, pH 7.0. We administered the pharmacological agents through the intraplantar injections by an established protocol for the purpose of reducing the inflammatory responses [62]. In brief, under ether anesthesia, a 26-gauge needle connected to a 10 μL Hamilton syringe (model: 701; Hamilton Company, Reno, NV, USA) was subdermally inserted into the plantar aspect of the ipsilateral side of injury. A volume of 10 μL pharmacological agents per rat was slowly injected for about 30 s.

#### 4.5.2. Behavioral Assessments

In this evaluation, we first established the behavioral assessments prior to CCI and recorded the neuropathic pain behaviors at POW 2 after CCI that were defined as PIH 0. Based on the concentrations of MPEP, we randomly separated into 500 mM, 250 mM, and vehicle (*n* = 6 per group). After a single intraplantar injection, each rat was measured every 1 h from 1 to 6 h (PIH 1 to PIH 6) and every 72 h from 24 to 168 h (PIH 24, PIH 96, and PIH 168) to evaluate the peripheral hypersensitivities resulted from the MPEP or vehicle. Withdrawal responses per side were averaged and a difference score was calculated by subtracting the response of contralateral side from the response of ipsilateral side.

### 4.6. Immunohistochemistry of Sciatic Nerve

#### 4.6.1. Procedures

At the arranged time points of experiments, rats were anesthetized and sacrificed. The distal stumps of sciatic nerve were fixed in 4% ice-cold paraformaldehyde in 0.1 M phosphate buffer (PB) for another 6 h and then changed to 0.1 M PB for storage. Distal stumps of sciatic nerve were cryoprotected with 30% sucrose in 0.1 M PB for 1 week and longitudinally sectioned at 8 μm thick with a cryostat (CM1850; Leica, Wetzlar, Germany). Sequentially labeled sections were treated with 0.5% Triton X-100 in 0.5 M Tris buffer (Tris), pH 7.6, for 30 min and processed for immunostaining. Briefly, the sections were quenched with 1% H_2_O_2_ in methanol and blocked with 5% normal goat serum in 0.5% nonfat dry milk/Tris. The sections were incubated with the diluted rabbit polyclonal metabotropic glutamate receptor subtype 5 (mGluR5) (1:500; Millipore) for 18~22 h. After rinsing in Tris, the sections were incubated with biotinylated goat anti-rabbit IgG (1:100; Jackson ImmunoResearch Laboratories) for 1 h, and the avidin-biotin complex for another hour. 3,3'-diaminobenzidine (Sigma, St. Louis, MO, USA) demonstrated the reaction products.

#### 4.6.2. Quantitation

High-definition monochrome images with minimal loss of signal were photographed under an Olympus microscope (BH2; Olympus, Tokyo, Japan) with a digital camera at a magnification of 400× following a protocol from a published method [63]. The histological fields of image acquisition were standardized as follows. In brief, a field equating to the midpoint of distal nerve stumps was first identified. From this position, the other two fields were sequentially identified across the nerve width in one nerve section. Adjacent fields in each nerve section were photographed ensuring that there was no partial cover of measured fields. All the areas-of-interest were measured with Adobe Photoshop Elements 2.0 (Adobe Systems) and edited above the threshold levels to exclude background noise. Each area-of-interest was measured in pixels and then transformed to μm^2^ according to the relationships between the pixel size and magnification. There were five nerve sections for each rat, and the investigator carrying out these measurements was blinded to the treatment of animals.

### 4.7. Double-Labeled Immunofluorescence

The sections from the distal stumps of sciatic nerve were processed for the double-labeled immunofluorescence. In brief, the sections were blocked with 5% normal goat serum with 0.5% Triton X-100 in 0.5 M Tris buffer (Tris) for 1 h at room temperature and incubated with one of a mixture of primary antiserum at 4 °C overnight: (1) mouse monoclonal SMI-31 (1:100; Sternberger Monoclonals Inc., Lutherville, MD, USA)/rabbit polyclonal mGluR5 (1:100; Millipore); (2) mouse monoclonal IB_4_ (1:100; Vector Laboratories)/rabbit polyclonal mGluR5 (1:100; Millipore); and (3) mouse monoclonal p75NTR (1:100; Millipore)/rabbit polyclonal mGluR5 (1:100; Millipore). After rinsing in Tris, sections were incubated with a mixture of secondary antibodies for another hour, *i.e.*, (1) fluorescein isothiocyanate (FITC)-conjugated anti-mouse immunoglobulin G (IgG) and (2) Texas Red-conjugated anti-rabbit IgG (both 1:100 and purchased from Jackson ImmunoResearch Laboratories). The sections were then dehydrated with 50% and 100% glycerol, mounted in VectoShield (Vector Laboratories), covered with a coverslip, and photographed under a conventional epifluorescent microscope (Zeiss Axiophot, Carl Zeiss, Heidelberg, Germany) equipped with appropriate filters.

### 4.8. Study Designs

To investigate the temporal effects of nerve compression, there were four groups of rats following CCI. The assessments for the altered neuropathic pain behaviors, including the thermal hyperalgesia and mechanical allodynia, were evaluated for all the rats at the following time points: The pre-test of baseline data before the surgery (designated POW 0), POW 1, POW 2, and POW 4. Then, the ipsilateral sides of rats at POW 0 were given the sham-operated surgery and sacrificed at POW 1 for morphological analysis (*n* = 5). The others after CCI were sacrificed at the following time points: POW 1, POW 2, and POW 4 (*n* = 5 per time point). Other rats with the surgery of TL were only evaluated and sacrificed at POW 1 (*n* = 5).

### 4.9. Statistical Analyses

Examiners were blinded to the grouping information when performing all the laboratory procedures of measurement and quantitation. Behavioral assessments and quantitations of IR area were presented as the mean ± standard deviation (SD), with GraphPad Prism (GraphPad, San Diego, CA, USA). Statistics obtained from behavioral responses and morphological examinations at the same time points were performed by student’s *t* test. For data that did not follow a Gaussian distribution, a nonparametric Mann-Whitney *U* test was conducted. *p* < 0.05, indicated a significant difference compared to the contralateral sides. The temporal changes of behavioral assessments were analyzed by two-way repeated measures ANOVA following Bonferroni’s *post-hoc* test when *p* < 0.05 was obtained. To analyze the within-group behavioral differences after pharmacological interventions, one-way repeated measures ANOVA was performed following Dunnett’s multiple comparison tests when *p* < 0.05 was obtained.

## 5. Conclusions

Through morphological analysis to understand behavioral variability revealed the following new findings with regard to a role of primary afferents after CCI: (1) we identified the progressive decreases of NF-200- and CGRP-IR SENFs, exhibiting partial cutaneous denervation, is an essential criterion to initiate thermal hyperalgesia and mechanical allodynia; (2) we further observed the intense increases of mGluR5-IR SENFs and proved the function of mGluR5 in periphery through the dose-dependently MPEP inhibitions; and (3) we confirmed that increased p75NTR-IR reactive Schwann cells surrounded the increased mGluR5-IR SENFs, which are indicating the nerve demyelination along the distal CCI stumps of sciatic nerve. In conclusion, our observations suggested the increases of mGluR5 expression in injured primary afferents resulted in developing peripheral hypersensitivities following CCI.
